# Dual RNA-seq in Streptococcus pneumoniae Infection Reveals Compartmentalized Neutrophil Responses in Lung and Pleural Space

**DOI:** 10.1128/mSystems.00216-19

**Published:** 2019-08-13

**Authors:** Neil D. Ritchie, Tom J. Evans

**Affiliations:** a Institute of Infection, Immunity and Inflammation, University of Glasgow, Glasgow Biomedical Research Centre, Glasgow, United Kingdom; University of Southampton

**Keywords:** transcriptomics, dual RNA-seq, neutrophils, pneumonia

## Abstract

Streptococcus pneumoniae is the dominant cause of community-acquired pneumonia worldwide. Invasion of the pleural space is common and results in increased mortality. We set out to determine the bacterial and host factors that influence invasion of the pleural space. In a murine model of pneumococcal infection, we isolated neutrophil-dominated samples of bronchoalveolar and pleural fluid containing bacteria 48 hours after infection. Using dual RNA sequencing (RNA-seq), we characterized bacterial and host transcripts that were differentially regulated between these compartments and bacteria in broth and resting neutrophils, respectively. Pleural and lung samples showed upregulation of genes involved in the positive regulation of neutrophil extravasation but downregulation of genes mediating bacterial killing. Compared to the lung samples, cells within the pleural space showed marked upregulation of many genes induced by type I interferons, which are cytokines implicated in preventing bacterial transmigration across epithelial barriers. Differences in the bacterial transcripts between the infected samples and bacteria grown in broth showed the upregulation of genes in the bacteriocin locus, the pneumococcal surface adhesin PsaA, and the glycopeptide resistance gene *vanZ;* the gene encoding the ClpP protease was downregulated in infection. One hundred sixty-nine intergenic putative small bacterial RNAs were also identified, of which 43 (25.4%) small RNAs had been previously described. Forty-two of the small RNAs were upregulated in pleura compared to broth, including many previously identified as being important in virulence. Our results have identified key host and bacterial responses to invasion of the pleural space that can be potentially exploited to develop alternative antimicrobial strategies for the prevention and treatment of pneumococcal pleural disease.

**IMPORTANCE** The factors that regulate the passage of bacteria between different anatomical compartments are unclear. We have used an experimental model of infection with Streptococcus pneumoniae to examine the host and bacterial factors involved in the passage of bacteria from the lung to the pleural space. The transcriptional profile of host and bacterial cells within the pleural space and lung was analyzed using deep sequencing of the entire transcriptome using the technique of dual RNA-seq. We found significant differences in the host and bacterial RNA profiles in infection, which shed light on the key factors that allow passage of this bacterium into the pleural space.

## Abstract (precis)

The factors that regulate the passage of bacteria between different anatomical compartments are unclear. We have used an experimental model of infection with Streptococcus pneumoniae to examine the host and bacterial factors involved in the passage of bacteria from the lung to the pleural space. The transcriptional profile of host and bacterial cells within the pleural space and lung was analyzed using deep sequencing of the entire transcriptome using the technique of dual RNA-seq. We found significant differences in the host and bacterial RNA profiles in infection, which shed light on the key factors that allow passage of this bacterium into the pleural space.

## INTRODUCTION

Streptococcus pneumoniae is the commonest cause of bacterial pneumonia and a significant cause of mortality worldwide (1, 2). The organism initially colonizes the nasopharynx from where it can invade other body sites (3). Spread to the lungs results from microaspiration and will result in pneumonia if not subjected to immune clearance. The infectious focus within the lungs produces an increase in pulmonary interstitial fluid and an increase in capillary permeability, leading to increased flow of fluid into the pleural space (4, 5). This commonly can result in a parapneumonic pleural effusion, which develops in about half of patients with pneumonia (6–8). Although prompt antibiotic treatment can limit the development of such pleural inflammation, continued inflammation and infection can result in a complicated parapneumonic effusion (a category 3 effusion), characterized by increased invasion of neutrophils with bacteria and the activation of the clotting cascade (8, 9). Such effusions will require a drainage procedure. If untreated, the effusion can then develop into the presence of frank pus and significant tethering adhesions within the pleural space, an empyema. About 10% of patients with pneumonia may develop an empyema, which may require open surgical drainage and has a significant mortality of up to 30% (7).

The host and bacterial factors dictating the development of empyema are not clear. Pneumococcal capsular serotypes 1, 3, and 19A are most commonly associated with complicated pleural disease in numerous studies carried out worldwide (10). Animal models have been utilized to attempt to define better the key factors involved in pleural space invasion. A rabbit model of pleural infection using intrapleural instillation of Pasteurella multocida found that increased levels of transforming growth factor-β correlated with pleural fibrosis, and that neutralization of this factor by specific antibody could attenuate this process (11, 12). More recently, using a mouse model of experimental empyema resulting from intranasal inoculation of S. pneumoniae, Wilkosz et al. found rapid bacterial invasion of the pleural space, with raised levels of interleukin 8 (IL-8), vascular endothelial growth factor (VEGF), monocyte chemoattractant protein 1 (MCP-1), and tumor necrosis factor alpha (TNF-α) (13). The pleural compartment offered a protective space for the bacteria. Migration of pneumococci across cellular barriers has been reported in a number of studies. The microbe has been shown to adhere to the platelet activating factor (PAF) receptor, which enhances migration across epithelial and endothelial barriers (14). The PAF receptor is downregulated by type I interferons during experimental pneumococcal infection, and these interferons also upregulate tight junction proteins (15). Transmigration of pneumococci into blood in this study was attenuated by exogenous β interferon, which also enhanced survival after intranasal pneumococcal infection. This correlates with other studies that have shown protective effects of type I interferon in pneumococcal infection (16, 17).

The aim of this investigation was to understand more clearly the host and bacterial factors that influence invasion of the pleural space in pneumococcal pneumonia. We used a murine model of infection with intranasal instillation of a type 3 pneumococcus which produced a lobar pneumonia and pleural invasion of bacteria. We examined the transcriptional responses of both bacteria and the neutrophils in lung and pleural space using the technique of dual RNA sequencing (RNA-seq). Compared to resting neutrophils, neutrophils in pleura showed upregulation of genes involved in migration but downregulation of genes mediating bacterial killing. Comparing the responses of neutrophils recovered from pleura with those from the lung showed a significant upregulation of type I interferon-inducible genes in cells recovered from the pleural space. Compared to bacteria grown in broth, relatively few genes were upregulated in the lung and pleura; these included members of the bacteriocin locus and the pneumococcal surface adhesin A *psaBCA* operon. We also identified bacterial transcripts of small RNAs (sRNAs), including putative novel sRNAs, many of which showed differential expression between bacteria isolated from pleura and those grown in broth.

## RESULTS

### Murine model of pneumococcal infection and isolation of RNA.

Mice were infected intranasally with a type 3 strain (sequence type 180 [ST180]) of S. pneumoniae (SRL1), and 48 h after infection, cells and bacteria were recovered from the pleural space and bronchoalveolar lavage fluid (BALF). The infected animals showed a progressive decline in weight and increase in a severity score, with a robust bacterial burden (>10^5^ CFU/ml of fluid) within pleural fluid and bronchoalveolar lavage fluid at this time, as well as penetration into the blood (see Fig. S1 in the supplemental material). The mean neutrophil percentages of bronchoalveolar and pleural fluids were 98.0% (*n* = 3; standard deviation [SD], 0.82%) and 99.0% (*n* = 3; SD, 0%), respectively, as assessed by differential counts from hematoxylin and eosin (H&E)-stained cytospin samples. Total RNA was isolated from pleural and bronchoalveolar lavage fluid, as well as from bacteria grown to mid-log phase in broth, and subjected to directional paired-end sequence analysis, as described in Materials and Methods. The quality of RNA from these samples was assessed using an Agilent 2100 Bioanalyzer. In general, the samples showed considerable degradation, with RNA integrity values between 2 and 3.1, despite repeated attempts at purification. This was thought to be due to the likely action of RNases within the infected inflammatory infiltrate. However, we proceeded with rRNA depletion and library preparation, which did produce adequately sized libraries. We used total RNA to make libraries since we wished to quantify both eukaryotic and bacterial transcripts; total RNA-based transcriptomes are also relatively free from artefacts in expression levels that may be caused by degraded RNA (18). Infected samples yielded 46.6 to 166 million reads per sample (median, 59.4 million); samples from bacteria grown in broth yielded 15.1 to 62.0 million reads (median, 57.8 million).

10.1128/mSystems.00216-19.1 FIG S1 Comparison of the strain SRL1 to the reference ST180 pneumococcus OXC141. The sequence read depth of SRL1 is shown at the top, with oligonucleotide variants per 10 kb shown at the bottom of the plot in red. Significant variations are marked below the plot and summarized in the table. Borders between shades of blue in the plot represent the minimum, median, and maximum read depth from bottom to top. Download FIG S1, PDF file, 0.8 MB. Copyright © 2019 Ritchie and Evans. 2019 Ritchie and Evans This content is distributed under the terms of the Creative Commons Attribution 4.0 International license.

The serotype 3 strain used, SRL1, was subjected to whole-genome sequencing. We obtained 21,632,672 reads, of which 99.52% mapped to the ST180 reference genome OXC141, to an average read depth of 1,039×. The genomes were very similar, as shown in Fig. S2. Ninety-two nonsynonymous sequence variations were present. Other structural variations are shown in Fig. S2; the most significant was the loss of the prophage sequence ΦSpn_OXC in the SRL1 sequence compared to OXC141. We analyzed the 48,703 reads from SRL1 that did not map to OXC141 to ensure we would capture all transcripts from this strain. The unmapped reads were assembled using SPAdes (19), which yielded a total of 120 scaffolds. Seven of these were greater than 500 bp in length; BLAST analysis revealed that none of these were from any putative streptococcal source. Metagenomics analysis of the unmatched reads using metaphlan (20) showed 85% of microbial reads were from viral sources and 15% were from Pseudomonas aeruginosa; these reads presumably reflect very low-level contamination of broth or reagents with nonviable organisms. Taken together, these results demonstrate that OXC141 provides an excellent reference to map SRL1 transcripts, with no evidence of significant genes in SRL1 that are not contained in OXC141.

10.1128/mSystems.00216-19.2 FIG S2 The course of bacterial infection in the dual RNA-seq experiment. (A) Change in body weight. (B) Clinical score. (C) Bacterial burden. Download FIG S2, PDF file, 0.06 MB. Copyright © 2019 Ritchie and Evans. 2019 Ritchie and Evans This content is distributed under the terms of the Creative Commons Attribution 4.0 International license.

Reads were aligned to murine and pneumococcal reference sequences, as described in Materials and Methods, and the read counts for genes were determined and further analyzed (Table 1).

**TABLE 1:** Read statistics for the different sequenced RNA samples matched to the reference type 3 pneumococcus sequence OXC141 or to the murine genome^*a*^

Sample by count type	Total no. of reads	No. of paired reads aligned	No. of single reads aligned	Total no. of aligned reads	% reads aligned
Bacterial					
Broth 1	61,987,154	44,551,264	5,247,430	49,798,694	80.4
Broth 2	57,882,676	31,983,782	4,008,135	35,991,917	62.0
Broth 3	15,145,016	10,084,854	2,137,236	12,222,090	80.3
BALF 1	46,267,280	7,098	4,798	11,896	<0.1
BALF 2	60,494,608	6,061,080	702,159	6,763,239	11.2
BALF 3	58,380,798	102,326	16,771	119,097	0.2
Pleura 1	57,787,368	272,956	41,306	314,262	0.5
Pleura 2	166,408,846	2,941,674	344,923	3,286,597	2.0
Pleura 3	62,976,262	82,604	31,310	113,914	0.18
Murine					
BALF 1	46,267,280	23,216,370	2,728,005	25,944,375	56.1
BALF 2	60,494,608	26,797,294	3,392,368	30,189,662	50.0
BALF 3	58,380,798	28,988,550	3,704,168	32,692,718	56.0
Pleura 1	57,787,368	28,491,010	5,585,637	34,076,647	59.0
Pleura 2	166,408,846	87,343,504	10,292,663	97,636,167	58.7
Pleura 3	62,976,262	34,412,624	6,047,740	40,460,366	64.2

a Reads were mapped to gene regions only.

A comparison of the compositions of different samples for both murine and bacterial gene counts showed considerable variation in the aligned bacterial reads in pleural fluid and BALF samples (range, <0.1 to 11.2% of total reads), while the reads aligned to the murine genome were much less variable (50.0 to 64.2% of total reads). Studies of read depth in a bacterial genome required to allow successful identification of transcripts depend on expression level but demonstrate that 100,000 reads are sufficient to identify genes expressed at levels of >10 reads per kilobase of transcript per million (RPKM) mapped reads, and even lower levels were sufficient to identify the majority of differentially expressed genes in an animal model of bacterial infection (21). On that basis, given the low number of aligned reads from bacterial counts in BALF sample 1, these were excluded from further analysis. The read counts for murine transcripts were more consistent and all were in excess of 25 million. The predominant cell type in the pleural and BALF samples was neutrophils. BALF and pleural washes from uninfected mice contain essentially no neutrophils. Thus, to allow comparison to resting neutrophils not exposed to an infectious agent, we compared read counts in our infected samples with a validated reference data set from resting bone marrow-derived neutrophils of C57BL/6 mice (22). Bacterial read counts were filtered to keep only those transcripts which were expressed at greater than 0.5 counts per million in at least 2 of the libraries. This ensured that genes with consistently low counts were excluded from analysis, as these are unlikely to give statistically reliable differential expression results. The murine gene read counts were filtered to keep transcripts with read counts of greater than 0.5 counts per million in at least 2 of the samples from the resting neutrophil libraries. This ensured that reads not significantly expressed within neutrophils were excluded, thus ensuring that potential reads from other cell types in the pleural fluid and BALF samples did not contribute to the analysis.

**FIG 1:**
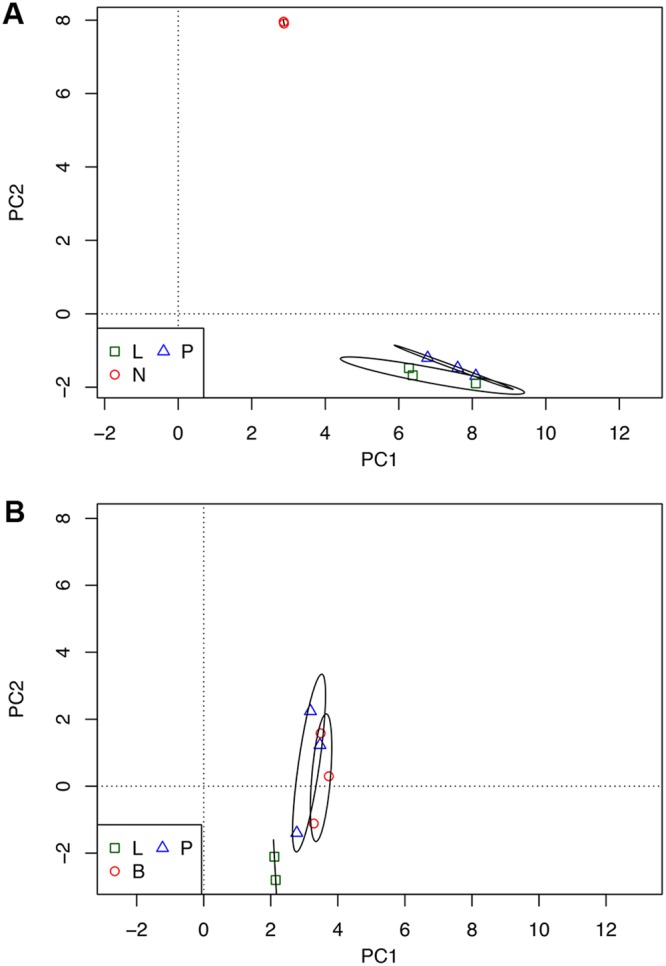
Principal-component analysis of the transcriptional profile of host and bacterial cells before and after infection. (A and B) Graphs plot the two largest principal components of the normalized transcriptome profiles of host (A) and bacterial (B) cells. Ellipses surround the 95% confidence limits of the centroids of the groups. L, lung samples; P, pleural samples; N, resting neutrophil samples; B, broth samples.

Overall differences between expression levels between the various samples were analyzed by principal-component analysis. Replicate samples of the murine read counts showed a clear separation between the resting neutrophil samples and the pleural fluid and BALF samples (Fig. 1A). The difference between the groups was significant (*P* < 0.005, permutational multivariate analysis of variance [PERMANOVA]). Overall differences in expression levels between the bacterial counts showed considerable overlap between samples from broth, pleura, and BALF (Fig. 1B); differences were not significant (*P* > 0.05, PERMANOVA).

### Unmatched reads.

As is seen in Table 1, a significant proportion of the sequenced reads did not match mouse or OXC141 gene regions. Further analysis matching to murine intergenic regions showed that about another 30% mapped to these areas, which was as expected since the RNA used for the analysis was total RNA and thus contains a variety of intergenic transcripts, as has been previously described (23, 24). Reads that did not map to either the complete murine genome or to the OXC141 genome were analyzed for their microbial content using metaphlan and assembled using rnaSPAdes (25). This revealed putative transcripts from a variety of murine viruses; the only significant bacterial component was from Escherichia coli, which varied between 0.05% and 0.61% of reads. As culture of BALF or pleural fluid never contained any E. coli cells, the significance of these reads is not clear. They may represent low levels of dead or dying organisms which are known to be part of the microbiome of the lower airways (26). Taken together, however, these analyses confirm that we had not missed significant numbers of SRL1 transcripts in our mapping to OXC141, in agreement with the data from the genomic sequencing.

### Differential gene expression.

**FIG 2:**
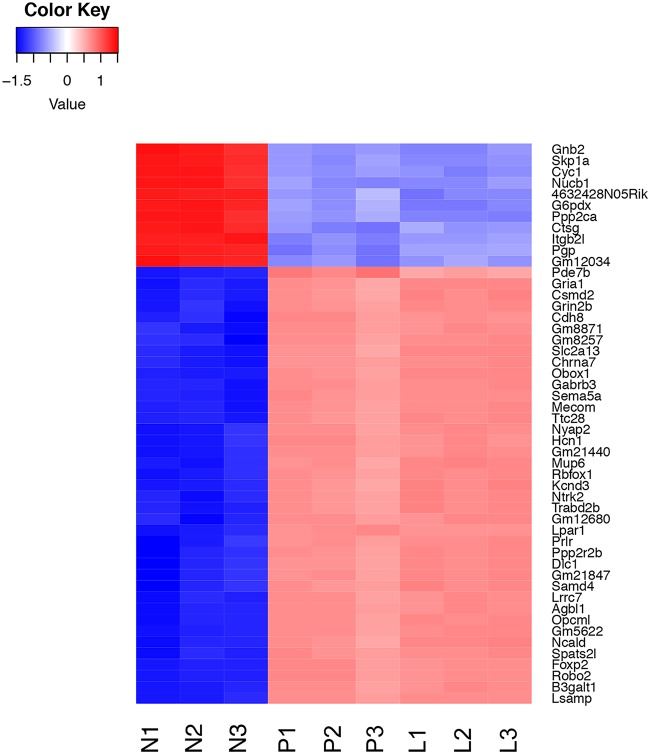
Heat map showing levels of expression of the top 50 differentially expressed host genes. Color coding shows the z score values of each sample as indicated in the scale, with red above the mean and blue beneath. Samples are coded as in Fig. 1.

**(i) Murine transcripts.** Murine transcripts were analyzed for differential expression between the different samples, as described in Materials and Methods. First, we compared differentially expressed transcripts between the pleural samples and the resting neutrophils. We examined genes that showed a greater than 2-fold change, with a false-discovery rate cutoff of 5%. This identified 4,394 genes that were upregulated in the pleural and/or lung samples compared to neutrophil samples and 5,316 that showed downregulation. The vast majority of these differentially regulated genes compared to resting neutrophils were common to both the lung and the pleural samples, as shown in the Venn diagram in Fig. S3. The top 50 significantly differentially expressed genes between the pleural and resting neutrophils are shown as a heat map in Fig. 2. The figure demonstrates very consistent changes, either up or down, in the triplicate replicates. Forty-five of the top 50 differentially expressed genes between the pleural and neutrophil samples were common to the top 50 differentially expressed genes between the lung and neutrophil samples; 48 genes were in the top 100 of the lung/neutrophil set, and all 50 genes were in the top 200.

10.1128/mSystems.00216-19.3 FIG S3 (A and B) Venn diagram showing distribution of significantly upregulated (A) and downregulated (B) genes between pleural and lung samples. Download FIG S3, PDF file, 0.3 MB. Copyright © 2019 Ritchie and Evans. 2019 Ritchie and Evans This content is distributed under the terms of the Creative Commons Attribution 4.0 International license.

**FIG 3:**
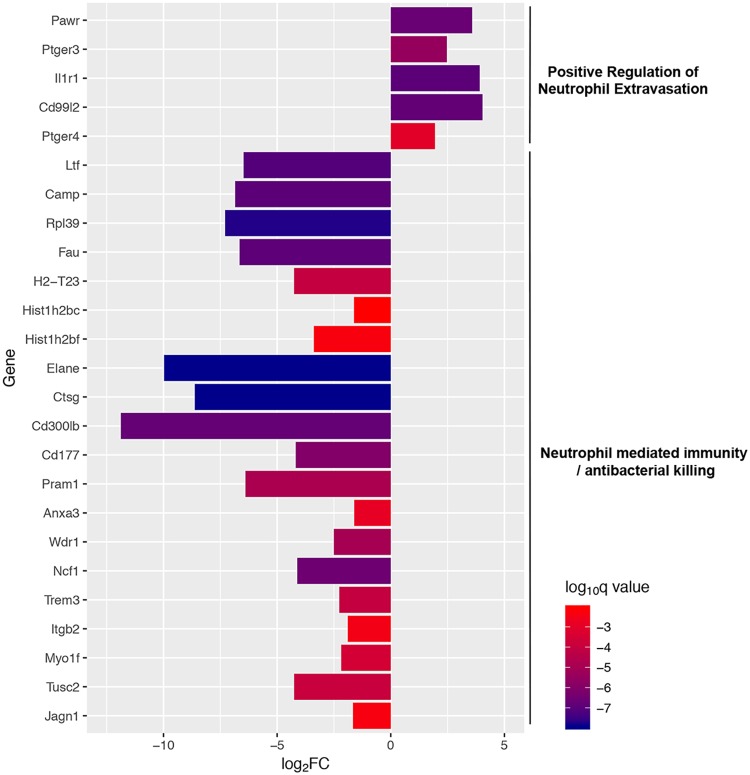
Differentially expressed genes between pleural and resting neutrophils. Graph shows the mean log_2_ fold change between pleural and resting neutrophils for the indicated genes. Genes are grouped according to the gene ontology terms shown to the right. Each bar is color coded according to the level of significance (*q* value) as shown by the scale bar.

In order better to understand the functions of these differentially expressed genes, we performed enrichment analysis of the gene ontology terms associated with these genes. Analysis of all significantly up- or downregulated genes between the pleural and neutrophil samples was not particularly informative (Fig. S4). For upregulated genes, the classes which were enriched were very broad and contained mostly terms related to neurological systems (Fig. S4A). The sets of significantly downregulated genes were more coherent, consisting of genes involved in general transcription and translation (Fig. S4B). To narrow down gene sets of interest, we focused on gene ontology terms containing the word neutrophil or bacterial killing. This identified two gene sets which were significantly underrepresented (neutrophil-mediated immunity and antibacterial humoral response) and one gene set which was overrepresented (positive regulation of neutrophil extravasation). The fold changes between the pleural and neutrophil samples of the individual genes contained in these different gene sets are shown in Fig. 3. Genes significantly upregulated within the positive regulation of neutrophil extravasation included CD99L, a murine homolog of human CD99 that is a key mediator of the transendothelial migration of neutrophils (27), and the IL-1 type 1 receptor, which mediates neutrophil migration induced by IL-1 (28). In contrast to the upregulation of genes involved in neutrophil migration, genes involved in immunity and bacterial killing were downregulated. These included the following genes directly mediating neutrophil killing: *Elane*, encoding neutrophil elastase; *Ltf*, encoding lactoferrin; *Camp*, encoding cathelicidin antimicrobial peptide; and *Ctsg*, encoding cathepsin G. These differences are considered further in the Discussion.

10.1128/mSystems.00216-19.4 FIG S4 (A and B) Gene ontology terms associated with host genes upregulated (A) and downregulated (B) in pleural neutrophils compared to resting cells. Each point represents the ratio of genes within the indicated gene ontology term to the total number of upregulated differentially expressed genes. The color of the points reflects the adjusted significance level (p.adjust) by the Benjamini-Hochberg method, as indicated by the scale. The size of the point reflects the number of genes represented by the point (count) as indicated. Download FIG S4, PDF file, 0.5 MB. Copyright © 2019 Ritchie and Evans. 2019 Ritchie and Evans This content is distributed under the terms of the Creative Commons Attribution 4.0 International license.

**FIG 4:**
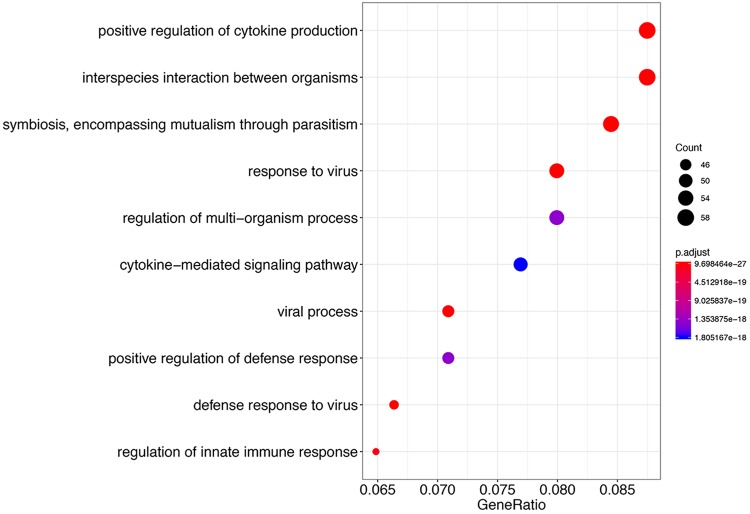
Gene ontology terms associated with host genes upregulated in pleura compared to the lung. Each point represents the ratio of genes within the indicated gene ontology term to the total number of upregulated differentially expressed genes. The color of the points reflects the adjusted significance level (p.adjust) by the Benjamini-Hochberg method, as indicated by the scale. The size of a point reflects the number of genes represented by the point (count) as indicated.

**FIG 5:**
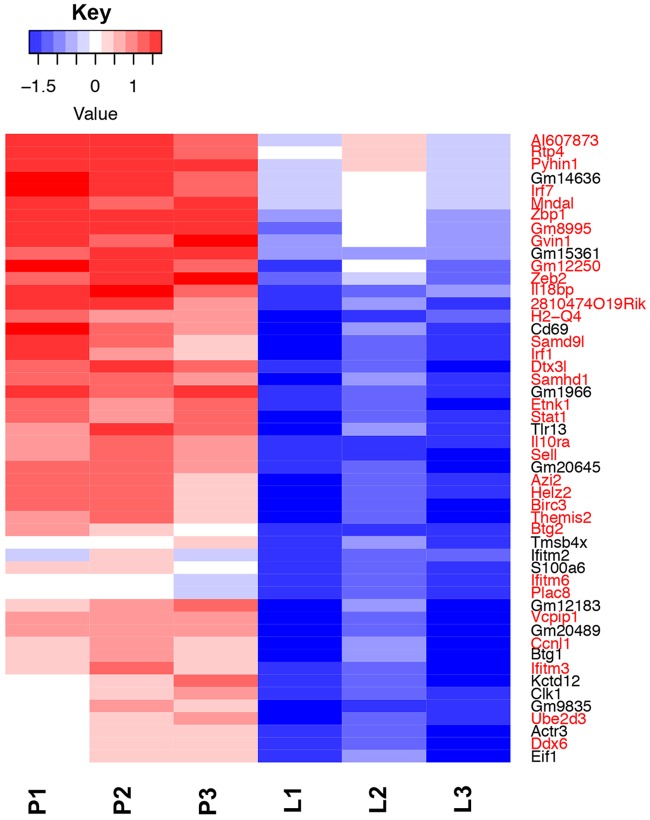
Heat map showing levels of expression of the top 50 differentially expressed host genes between pleural (P) and lung (L) samples. Color coding shows the z score values of each sample, as indicated in the scale, with red above the mean and blue beneath. Genes highlighted in red belong to the set of genes induced by type I interferons.

Next, we determined murine genes that were differentially expressed between the pleural and lung neutrophil samples. We identified 132 genes that were upregulated in the pleural samples compared to the lung; no genes were downregulated. Gene enrichment analysis of these gene sets is shown in Fig. 4. These included genes involved in cytokine production and immune response to viruses. A heat map of the top 50 differentially expressed genes between the pleura and lung is shown in Fig. 5. Although there is some variation between samples, the overall patterns between the triplicate samples are consistent. A notable feature of this gene set is the high number of genes which are regulated by type I interferons, comprising 33 out of these top 50 genes (shown in red in Fig. 5) (29). Compared to the 1,925 type I interferon-regulated genes out of 22,971 protein-coding genes within the mouse genome, this is a highly significant enrichment (*P* = 2.4 × 10^−25^, hypergeometric test). The enrichment of type I interferon-regulated genes in the pleural samples is considered further in the Discussion.

**FIG 6:**
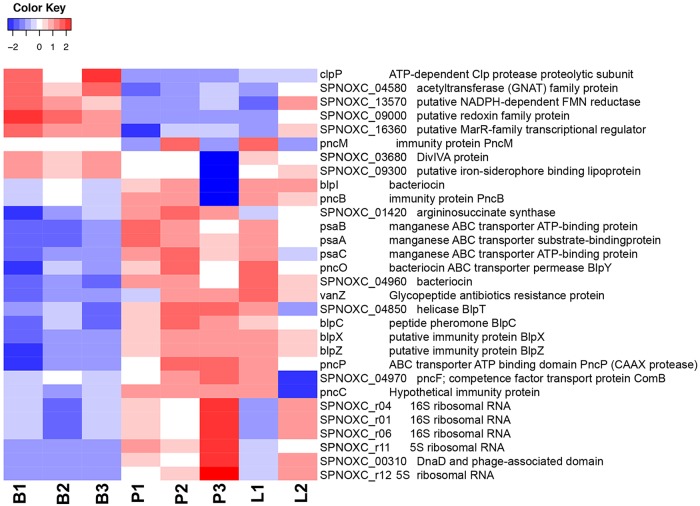
Heat map showing levels of expression of the top 30 differentially expressed bacterial genes between broth (B), pleura (P), and the lung (L). Color coding shows the z score values of each sample as indicated in the scale, with red above the mean and blue beneath. GNAT, Gcn5-related *N*-acetyltransferase; FMN, flavin mononucleotide.

**(ii) Bacterial transcripts.** We compared bacterial transcripts present in pleural fluid and BALF with those present in the same bacteria grown in broth. There will of course likely be genes expressed in broth culture that are important in causing disease. However, bacteria isolated from any site of an infected mouse will also likely have expression of genes important in infection in general. A comparison of transcripts from pleural fluid and BALF invasive bacteria to those in broth will detect transcripts that are not required for growth under an unstressed condition in a rich nutrient broth. We identified bacterial genes that were significantly differentially regulated between pleural and broth samples with a log_2_ fold change of 1.5. This identified 8 genes that were downregulated in pleura compared to broth and 22 genes that were upregulated. These are shown in the heat map in Fig. 6. There were no significantly differentially expressed genes between the pleural and lung samples. The upregulated genes belonged to three main groups. First, there are a number of genes contained within the *blp* cassette, also known as the bacteriocin immunity region. These included the *blpC* gene encoding BlpC, the peptide pheromone that initiates transcription from the whole region, as well as genes encoding immunity proteins, *pncB*, *pncO*, *pncP*, *blpX*, *blpY,* and *blpZ*, and a gene encoding a protein of unknown function, *blpT* (30, 31). Sequence analysis of the bacteriocin immunity region of the ST180 strain used in these experiments shows that it contains a common frameshift mutation within the *blpA* gene that encodes the BlpA component of the BlpAB transporter, a transmembrane complex that acts as a conduit for the secretion of BlpC and bacteriocins. This results in a premature termination of the *blpA* product and a nonfunctional BlpAB transporter. Recent work has shown that BlpC and bacteriocins can also be exported by the ComAB transporter that mediates export of the ComC peptide, a precursor of the competence-stimulating peptide (32–34). The upregulation of the *blp* locus seen in our experiments with the ST180 strain demonstrates that this competence transporter system is active during *in vivo* infection and allows the export of BlpC and activation of bacteriocin-related genes. We also found that the gene for the ComB component of the ComAB transporter was significantly upregulated in the pleural samples.

A second set of genes upregulated in the pleural and lung infected samples were the *psaA*, *psaB,* and *psaC* genes, elements of the *psaBCA* operon which encodes the PsaA pneumococcal surface adhesins, a manganese binding ABC-type lipoprotein, and the associated proteins PsaB and PsaC (35). PsaA acts as an adhesion, binding to E-cadherin in epithelial surfaces (36), is a known virulence determinant in invasive pneumococcal disease (37) and was identified as essential for growth in human pleural fluid *in vitro* (38). A third set of upregulated genes in the pleural and lung samples were those encoding structural RNA components of the ribosome, suggesting increased protein expression under conditions of fast growth in the pleura and lung compared to the growth in mid-log phase in broth (39).

Finally, we noted significant upregulation of the *vanZ* gene, encoding a glycopeptide antibiotic resistance protein, and a gene encoding arginosuccinate synthase. VanZ has previously been found to have a role in bacterial invasion of blood (40), but its molecular action is not clear. Arginosuccinate synthase has been linked to pneumococcal virulence and resistance to oxidative stress (41).

A number of genes were significantly downregulated in the infected samples compared to bacteria grown in broth. The gene *clpP* encoding the ClpP ATP-dependent protease was downregulated in the infected samples. ClpP has been implicated in bacterial virulence (42) and thermotolerance (43) but also as a negative regulator of the competence system (43, 44). From our results, it would seem that downregulation of ClpP is correlated with an upregulation in the competence system and not affecting virulence genes; this is considered further in the Discussion. The other downregulated genes have less clearly defined roles.

### Small RNAs expressed in bacteria grown in broth and from pleural and lung isolates.

Small bacterial RNAs are increasingly recognized as potentially important regulators of the bacterial transcriptome, particularly in adaptations that may be important in virulence (45). Thus, we interrogated our RNA-seq data to examine the expression level of putative small RNAs expressed in bacteria grown in broth compared to those isolated from the lung or pleura that might therefore be involved in adaptation to the anatomical locations. To identify putative sRNAs, we used the software system Rockhopper (46). In order to exclude partial and overlapping transcripts, we selected novel transcripts greater than 100 bp in length that did not correspond to known genes. In order to increase the specificity of identification of putative sRNAs, we then filtered these transcripts to those from intergenic regions by excluding transcripts that had greater than or equal to a 10-bp overlap with an annotated gene at either the 5′ or 3′ end. We identified 169 putative intergenic sRNAs using this method and compared expression levels between pleural samples and broth culture. The full details for these sRNAs are shown in Table S1. Forty-three (25.4%) of these transcripts corresponded to previously identified sRNAs from the S. pneumoniae strain TIGR4 (47–49), leaving 126 potentially novel sRNAs. sRNAs were evenly distributed between the positive and negative strands (Fig. S5). BLAST analysis of these sequences against the reference serotype 3 OXC141 genome showed that 113 (67%) of these sequences were unique, while the remaining 56 (33%) sequences were repeated many times in the genome (range, 2 to 86 sequences; median, 7 sequences).

10.1128/mSystems.00216-19.5 FIG S5 Mapping of small RNA positions onto OXC141 reference genome. From the outside in, the plots show the following: position on the genome, positive-strand genes, negative-strand genes, rRNA, tRNA, positive-strand sRNA, negative-strand sRNA, GC content, and GC skew. Download FIG S5, PDF file, 0.7 MB. Copyright © 2019 Ritchie and Evans. 2019 Ritchie and Evans This content is distributed under the terms of the Creative Commons Attribution 4.0 International license.

**FIG 7:**
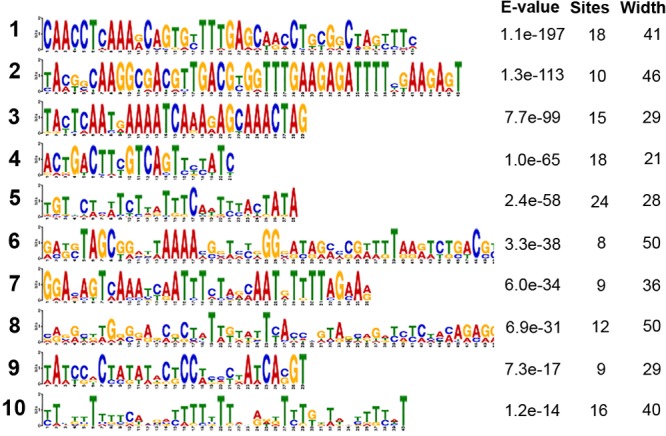
Motifs within the small bacterial RNAs generated by MEME. The sequence of each motif is shown, with the height of a nucleotide symbol indicating the degree of conservation at that position. The program was run to find a maximum of 10 motifs. The E value is an estimate of the expected number of motifs with the given log likelihood ratio (or higher), and with the same width and site count that one would find in a similarly sized set of random sequences (sequences where each position is independent, and letters are chosen according to the background letter frequencies). “Sites” indicates the number of occurrences within the small RNA set, and “Width” is the length of the motif.

10.1128/mSystems.00216-19.7 TABLE S1 sRNAs identified within bacterial RNA profiles isolated from pleura and broth. Table shows the location of the sRNA; polarity of transcription (strand); expression levels in pleura and broth (as reads per kilobase of transcript per million mapped reads); relative expression in pleura compared to broth; significance level of the expressed genes in pleural sample compared to broth (*q* value, corrected by the Benjamini-Hochberg method); length in bp; sequence; number of copies in SRL1 genome; RNA family member; overlap with sRNA from opposite strand; motif number within sRNA as shown in Fig. 7; free energy of folding; previous identification in papers by Mann et al. (49), Acebo et al. (48), and Kumar et al. (47); and role in virulence as found in Mann et al. (49). Download Table S1, XLSX file, 0.03 MB. Copyright © 2019 Ritchie and Evans. 2019 Ritchie and Evans This content is distributed under the terms of the Creative Commons Attribution 4.0 International license.

We sought to identify any conserved or repeated motifs within this collection of sRNAs using the MEME software. This identified a number of well-conserved sequence motifs; the 10 most significant consensus sequences are shown in Fig. 7, and their occurrences in the different sRNAs are indicated in Table S1. Some sRNAs carried more than 1 of these motifs, and some had repeats of a specific motif. None of the motifs shown in Fig. 7 show significant homology to the previously described motifs in pneumococcal sRNAs (49) or to the BOX, RUP, or SPRITE repeated elements that are dispersed through pneumococcal genomes (50). All of the sRNA sequences were analyzed for conserved noncoding RNA elements using the Rfam database (51). These are indicated in Table S1. These included the following: *cia*-dependent small RNA (csRNA), an sRNA family that is part of the CiaRH two-component regulatory system in S. pneumoniae that controls stationary-phase autolysis (52); transfer-messenger RNA (tmRNA), involved in processing stalled transcripts (53); group-II-D1D4-3, a mobile type II intron element (54); T-box, a riboswitch element (55); TPP, a thiamine-sensing riboswitch (56); and sRNAs in the Rfam database from the previous study by Mann et al. (49).

We selected sRNAs that were upregulated >2-fold in the pleural samples compared to broth, with adjusted *P* values of <0.05 (Table S2). Forty-two of the sRNAs fulfilled these criteria; 13 of these (31.0%) had been previously described, and a number were previously identified as playing a role in bacterial virulence. Some the identified putative sRNA contained more than 1 copy of previously identified sRNA sequences, e.g., sRNA120, which is highly copied in the OXC141 genome (71 copies) and has matches within the previously described sRNAs F29, F30, and F48 from Mann et al. (49). Many of these sRNAs were predicted to have a significant secondary structure. For example, the most highly differentially expressed sRNA in pleura, sRNA47, has a predicted stem-loop structure, as shown in Fig. S6. Many, but not all, sRNAs have a rho-independent terminator region; for example, the sRNA srn049 in the study by Acebo et al. (48) does not. No rho-independent terminator region is present in sRNA47 either. Further analysis of the role of this and the other sRNAs is ongoing to establish a definitive role in pleural and lung bacterial invasion.

10.1128/mSystems.00216-19.6 FIG S6 Model of secondary structure of small RNA number 47. Download FIG S6, PDF file, 0.03 MB. Copyright © 2019 Ritchie and Evans. 2019 Ritchie and Evans This content is distributed under the terms of the Creative Commons Attribution 4.0 International license.

10.1128/mSystems.00216-19.8 TABLE S2 sRNAs from Table S2 that are significantly differentially expressed between pleura and broth. Download Table S2, XLSX file, 0.01 MB. Copyright © 2019 Ritchie and Evans. 2019 Ritchie and Evans This content is distributed under the terms of the Creative Commons Attribution 4.0 International license.

## DISCUSSION

We have utilized dual RNA-seq to interrogate the host and bacterial responses to experimental pneumococcal infection within the lung and pleural space. We have shown that the neutrophil host response to infection in lung and pleural space involves up- and downregulation of common large sets of genes compared to resting neutrophils. Far fewer genes were differentially regulated between cells in pleural space compared to those in the bronchoalveolar fluid; these were dominated by genes upregulated in the pleural space by type I interferons. Changes in the bacterial transcriptional profile within the lung and pleural space compared to growth in broth were more limited. Notably, genes within the bacteriocin locus were significantly upregulated in infection, as was the known virulence determinant pneumococcal surface adhesin. A number of putative and known small RNAs were also defined in the bacterial transcripts, with differential expression of a number within infected cells in lung and pleura. This study thus provides important insight into the transcriptional programs that are activated in the host and bacterium upon invasion of the pleural space and has revealed new infection-specific transcripts that may contribute to bacterial invasion and host defense of the pleural space.

Dual RNA-seq of an *in vivo* infection provides important insights into host-pathogen interactions but presents a number of experimental challenges. Host cells will dominate the transcriptional profile, and a number of cell types will be present. We reduced variability from this factor by analyzing cells in bronchoalveolar lavage fluid and pleural space in our infection model, which are dominated by neutrophils. We also excluded transcripts that were present at <0.5 counts per million reads in at least 2 of the resting neutrophil samples, thus excluding RNA species that were not significantly expressed within neutrophils. Some transcripts will of course be found not only in neutrophils but in other cells; however, the dominance of the neutrophil response in the bronchoalveolar lavage fluid and pleural space will limit such contributions, and they are not likely to impact greatly the data presented here. Bacterial transcripts, as expected, formed only a small percentage of the total reads obtained, which will always be a challenge in *in vivo*. However, we were able to obtain sufficient reads from 5 of the 6 infected samples to identify transcripts with enough power to establish statistically significant patterns of differential expression. Transcripts expressed at lower levels may, however, be missed.

The host response to infection in the lung and pleural space neutrophils can be divided into the transcriptional response in these cells compared to resting neutrophils and the differential response between lung and pleural samples. Not surprisingly, there were large numbers of genes up- and downregulated in the lung and pleural samples compared to control resting neutrophils. Although neutrophils are relatively short-lived cells *in vivo*, they have a sophisticated transcriptional response to infectious or inflammatory stimuli that make important contributions to the host response to infection and inflammation (57, 58). Global analysis of patterns of differentially expressed genes was not particularly informative, but focusing on genes involved in neutrophil functions revealed a differential response. As would be expected from neutrophils recovered from bronchoalveolar lavage fluid or the pleural space, these cells showed upregulation of genes promoting neutrophil extravasation. However, genes involved in bacterial killing and immunity were at the same time downregulated; these included those encoding neutrophil elastase and other granule proteins. This may represent host-mediated downregulation of neutrophil inflammation to prevent tissue damage, or it might represent bacterially driven dampening of neutrophil killing as a virulence mechanism. Further experiments will be required to differentiate these two possibilities. We have previously shown the persistent presence of viable SRL1 bacteria within the pleural space at 48 h after infection despite a dense neutrophil infiltrate (59); downregulation of neutrophil antibacterial killing genes may account for this and will be the subject of further investigations. It is also worth highlighting that the strain used in these experiments (SRL1) is capsular serotype 3, a serotype that does have some unusual features. Serotype 3 strains have one of the highest degrees of encapsulation among the pneumococci (60), and the capsule is not covalently bound to the bacterium and is thus shed during growth (61). This serotype is carried in the nasopharynx at high prevalence but is less frequently isolated from blood cultures, yet it has one of the highest odds ratios of death in pneumonic bacteremic patients (60, 62). In our animal model of pneumococcal pneumonia, SRL1 produced a very marked lobar pneumonia, with relatively late invasion of blood compared to a serotype 4 strain with a lower degree of encapsulation (59). Thus, different serotypes may show different transcriptional responses from those reported here for serotype 3. This will likely reflect these significant differences in invasive potential and degree of encapsulation, which will influence the interaction between the bacteria and neutrophils.

A notable feature of the transcriptional response of the cells within the pleural space compared to those recovered from lung was the significant upregulation of a number of genes induced by type I interferons. As outlined in the introduction, type I interferons have been shown to be important in limiting the passage of pneumococci across epithelial barriers (15) and in the host defense against infection (17). Our data show that neutrophils within the pleural space, but not the lung, have been exposed to type I interferons. This suggests that a cell type or types within the pleural space are responsible for type I interferon production during pneumococcal infection that would be important in limiting bacterial entry into this space. Resident pleural macrophages have been shown to be important in neutrophil recruitment in inflammation (63) and would be a likely source, but further investigation will be required to establish the responsible cell type.

In contrast to the large-scale changes in neutrophil transcription in infection, the bacterial response in pleura and lung compared to cells grown in broth was quite limited. This may reflect the technical difficulties of obtaining sufficient bacterial reads from the *in vivo* samples and the relatively low sample sizes. Of the upregulated genes, significantly increased transcription from the bacteriocin locus was notable. The strain of pneumococcus used in our model has a commonly found missense mutation in the gene encoding the BlpA component of the key bacteriocin transporter BlpAB, a transmembrane complex that acts as a conduit for secretion of the inducing BlpC pheromone and bacteriocins (64). Although initially this was thought to prevent induction of the bacteriocin locus, subsequent investigation has revealed that at least *in vitro*, the ComAB transporter that mediates export of the ComC peptide, a precursor of the competence-stimulating peptide, can export BlpC and bacteriocins and circumvent this defect (32–34). Our work thus provides supporting *in vivo* evidence that bacteriocin production can be induced by this means, and it implies that competence is induced in bacterial cells in the lung and pleural space in this model of infection. This is also supported by the observed upregulation of the gene encoding the ComB component of the ComAB transporter in bacteria recovered from infected animals, and downregulation of the gene *clpP* encoding the ClpP ATP-dependent protease, which is a negative regulator of the competence system. Bacteriocin production and induction of competence have been shown to be important in the nasopharyngeal colonization phase of pneumococcal infection, mediating neighbor bacterial predation and the uptake of exogenous DNA, respectively. Competence systems are also upregulated in pneumococci exposed to cigarette smoke (65), and the bacteriocin locus has also been shown to be upregulated in bacteria dispersed by influenza A virus (66). Expression of bacteriocin genes within lung or pleural space was unexpected, since there would be minimal presence of other bacteria in these loci and hence no need for neighbor predation. This suggests that bacteriocins may be performing other functions during infection.

We identified 169 intergenic small transcripts, 43 of which corresponded to previously described sRNAs, leaving 126 potentially novel sRNAs. Definitive demonstration of these transcripts will require further work, but the high numbers of previously described sRNAs identified by our bioinformatics analysis of the RNA-seq data give us confidence that a significant proportion of this number will be genuine sRNAs. We also identified a number of known sRNAs as significantly upregulated in the pleural space compared to their expression in bacteria grown in broth. This includes a number previously identified as required for the invasion of specific body sites. Many of the putative sRNAs contain conserved motifs that are repeated within the SRL1 pneumococcal genome, as has been described for TIGR4 (49). Whether these sequences are important in the function of sRNAs is not clear.

In summary, our findings have revealed new aspects of the interactions between the pathogen S. pneumoniae and its host as the bacterium passes between the different anatomical sites of the lung and pleural space. We have shown that dual RNA-seq can yield useful data even with low bacterial numbers recovered from different sites in an *in vivo* model. We have identified key host and bacterial responses to invasion of the pleural space that have identified potential therapeutic targets for alternative treatments for invasive pulmonary pneumococcal disease.

## MATERIALS AND METHODS

### Bacterial strain.

A serogroup 3 pneumococcus, SRL1, was used in the experiments. It was obtained from the Scottish *Haemophilus, Legionella, Meningococcus,* and *Pneumococcus* Reference laboratory. It belongs to sequence type 180 (ST180). Bacteria were cultured on blood agar plates in an atmosphere of 5% CO_2_ and in liquid culture in brain heart infusion broth without agitation.

### Animal model of infection.

The animal model of infection used was as described by Ritchie et al. (59). We infected animals with 10^6^ CFU of SRL1 by the intranasal route. This dose reproducibly produces a lobar pneumonia with infiltration of the blood and pleural space by bacteria (59) and accumulation of neutrophils in the lung and pleural space. Three animals were culled at 48 h after infection. Bronchoalveolar lavage was performed as follows. Taking care to avoid puncturing the cervical vessels, the anterior neck was dissected down to expose the trachea and a small nick made in the anterior portion of the trachea to allow the insertion of a 1-ml fine-tip pastette containing 1 ml of ice-cold phosphate-buffered saline (PBS). PBS was carefully lavaged three times with collection back into the pastette. Cells from the pleural space were collected as follows. The anterior chest wall was dissected to expose the ribs. The abdomen was opened and the diaphragm exposed by carefully withdrawing the liver. The thoracic cavity was then infused through an exposed intercostal space with 5 ml of ice-cold PBS. The mouse was gently shaken to mix the fluid, and it was then aspirated via a transdiaphragmatic approach. Aliquots of these samples were suitably diluted and colony counts determined by a plate assay. The remainder of the sample was immediately added to 5 ml TRIzol reagent (Invitrogen, Thermo Fisher Scientific) with 1 ml of added 0.1-mm zirconia beads (Fisher Scientific) and processed as described below. Cell compositions of the pleural space and BALF were determined by triplicate cytospin preparation of samples which were stained with hematoxylin and eosin and examined by light microscopy.

### Ethics approval.

All animal work was carried out according to the Animals (Scientific Procedures) Act 1986 (ASPA). ASPA has recently been revised to transpose European Directive 2010/63/EU on the protection of animals used for scientific purposes. The work was approved by the UK Government Home Office under project license XC2FD842E.

### RNA extraction and purification.

Samples in TRIzol reagent with zirconia beads from the animal model of infection were vortexed for 10 min to ensure complete lysis. Bacteria were also grown to mid-log phase in broth and processed in an identical fashion. Chloroform (1.33 ml) was added to each tube before vigorous shaking and incubation for 10 min at room temperature. Samples were then centrifuged at 8,000 × *g* for 15 min at 4°C and the aqueous phase removed with a Pasteur pipette. RNA was extracted using the Zymo RNA Clean & Concentrator kit (Zymo Research), according to the manufacturer’s instructions. Further purification was performed with Agencourt RNAClean XP beads (Beckman Coulter). rRNA was depleted using the Ribo-Zero rRNA removal kit (Illumina), according to the manufacturer’s instructions. RNA concentration and quality were assessed using an Agilent 2100 Bioanalyzer.

### Library preparation and RNA sequencing.

Libraries were prepared from purified RNA using the strand-specific ScriptSeq library prep kit (Illumina) at the Centre for Genomic Research, University of Liverpool. Bacterial DNA was prepared using the Qiagen DNeasy blood and tissue preparation kit, according to the manufacturer’s instructions. DNA libraries were made using the Illumina TruSeq library prep kit. Libraries were sequenced using the HiSeq 2500 platform for 2 × 100-bp paired-end sequences; all 9 samples were sequenced in one lane, generating >270 million reads per lane. Reads were demultiplexed and adaptors and low-quality reads removed using Cutadapt and Sickle (minimum window quality score, 20), respectively.

### Bioinformatic analysis.

The experimental strain SRL1 was separately subjected to paired-end next-generation sequencing with 100-bp reads using a HiSeq 2500 platform at the Centre for Genomic Research, University of Liverpool, and reads were aligned to the reference genome OX141, a serotype 3 ST180 pneumococcus (67).

Trimmed reads were aligned to the OXC141 reference and mouse genomes using CLC Genomics Workbench (Qiagen). Raw read counts aligned to the bacterial and murine genomes are shown in Tables S3 and S4, respectively. Read counts assigned to genes were then analyzed using the edgeR pipeline (68). Principal-component analysis was performed using the function rda in the Vegan package in R (69). Ellipses surrounding the 95% confidence limits of the centroids of each group were created using the function ordiellipse in Vegan. Significantly differentially expressed genes were determined using the glmtreat function of edgeR with a 2-fold change in expression value; a false-discovery rate of 5% was used with the Benjamini-Hochberg method. Heat maps were prepared using the function heatmap.2 in the R package gplots. Overrepresentation of gene ontology families in the differentially expressed genes was analyzed and visualized using the package clusterProfiler (70). sRNAs were identified in the reads using Rockhopper 2 (71) and filtering on reads greater than 100 bp in length not assigned to a known gene. These reads were then compared to the gene locations from the reference genome OXC141 (GenBank accession no. NC_017592), and any transcripts that had a ≥10-bp overlap with the start or end of an annotated gene were removed. This left novel transcripts from intergenic regions only. Differential expression between the reads from different samples was calculated in Rockhopper with a 2-fold change in expression value; a false-discovery rate of 5% was used with the Benjamini-Hochberg method. Putative sRNAs thus identified from OXC141 were compared to the whole OXC141 and TIGR4 reference genomes using BLAST via the R interface rBLAST. Mapping of sRNAs to the OXC141 genome was visualized using DNAPlotter (72).

10.1128/mSystems.00216-19.9 TABLE S3 Read counts of bacterial transcripts aligned to SRL1 from the broth (B), pleural (P), and BALF (L) samples. Download Table S3, XLSX file, 0.1 MB. Copyright © 2019 Ritchie and Evans. 2019 Ritchie and Evans This content is distributed under the terms of the Creative Commons Attribution 4.0 International license.

10.1128/mSystems.00216-19.10 TABLE S4 Read counts of murine transcripts aligned to the mouse genome from neutrophil (N), pleural (P), and BALF (L) samples. Download Table S4, XLSX file, 2.5 MB. Copyright © 2019 Ritchie and Evans. 2019 Ritchie and Evans This content is distributed under the terms of the Creative Commons Attribution 4.0 International license.

Motifs repeated within the identified sRNAs were identified using MEME (73). RNA motifs within sRNAs were identified using Rfam (51). RNA secondary structure and free energies were calculated using Mfold (74).

Further analysis of unmapped reads was performed using SPAdes (19) and meatphlan2 (20), as described in the text.

### Data availability.

Reads from SRL1 aligned to OXC141 were deposited in the NCBI Sequence Read Archive under BioProject PRJNA553174, sample SRL1_ST180. Gene transcript counts are contained in Tables S1 to S4, and raw data with metadata were deposited in the Gene Expression Omnibus under accession number GSE134118.
