# Supplementary material for: Dual RNA-seq in Streptococcus pneumoniae Infection Reveals Compartmentalized Neutrophil Responses in Lung and Pleural Space
Source: mSystems. 2019 Aug 13;4(4):e00216-19. doi: 10.1128/mSystems.00216-19 (PMC6697439; doi:10.1128/mSystems.00216-19)
Supplement: FIG S1 [file mSystems.00216-19-sf001.pdf]

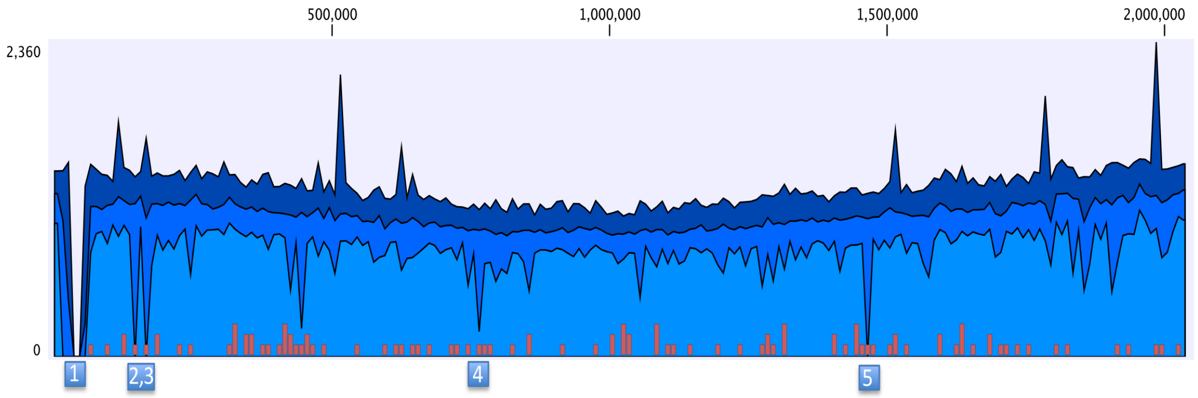

N	Description of variation compared to OXC141 genome
1	34kb deletion in SRL1 genome. Corresponds to putative pro-phage sequence. Sequence is entirely removed with cross mapped breakpoints at pro-phage insertion site.
2	Deletion involving a non-coding region
3	Poorly resolved area in highly repetitive region of PspA gene. Insufficient information to allow complete sequence to be determined.
4	Presence of a full copy of the glutamine ABC transporter permease gene. OXC141 has a deletion in this gene. The 285 bp insertion sequence was reconstructed using reads unmapped to the reference sequence and matched with 100% sequence homology to several published pneumococcal genomes including D39.
5	93 bp deletion in a peptide ABC transporter gene. Deletion is not present in any published pneumococcal sequences. Deletion is in-frame and affects the terminal region of the eflux pump. The functional consequence from this limited analysis is not clear.
