# Supplementary figures and images for: Dual RNA-seq in Streptococcus pneumoniae Infection Reveals Compartmentalized Neutrophil Responses in Lung and Pleural Space
Source: mSystems. 2019 Aug 13;4(4):e00216-19. doi: 10.1128/mSystems.00216-19 (PMC6697439; doi:10.1128/mSystems.00216-19)

A

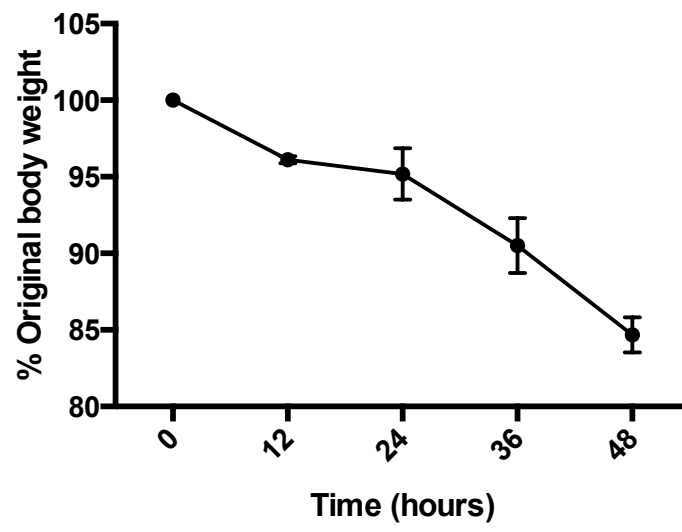

B

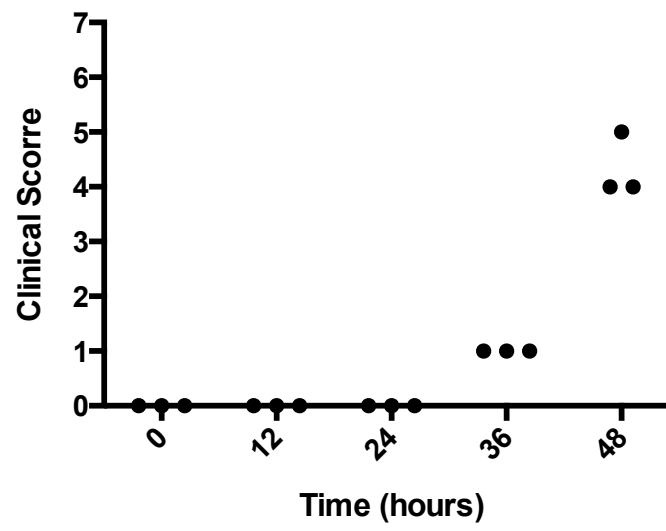

C

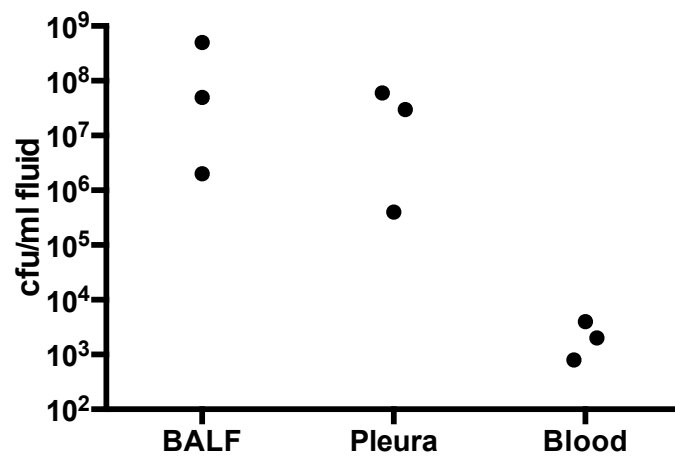

Supplement: FIG S2 [file mSystems.00216-19-sf002.pdf]

**A Upregulated**

Pleura

Lung

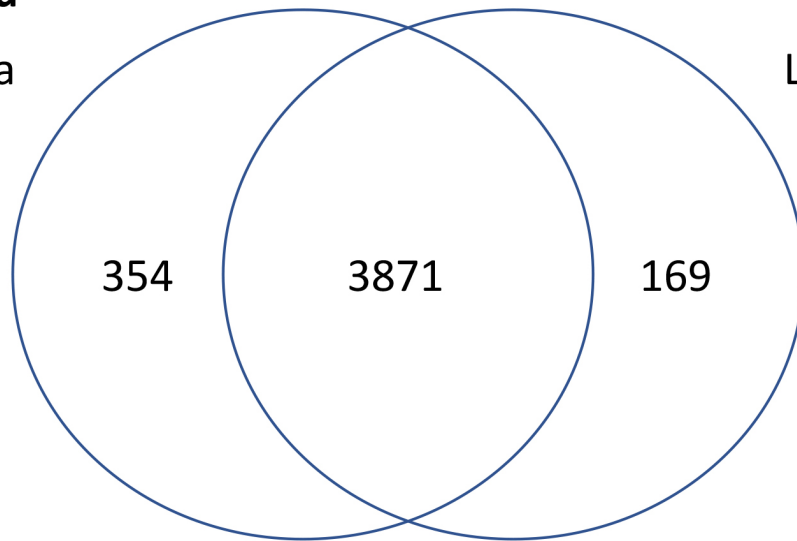

**B Downregulated**

Pleura

Lung

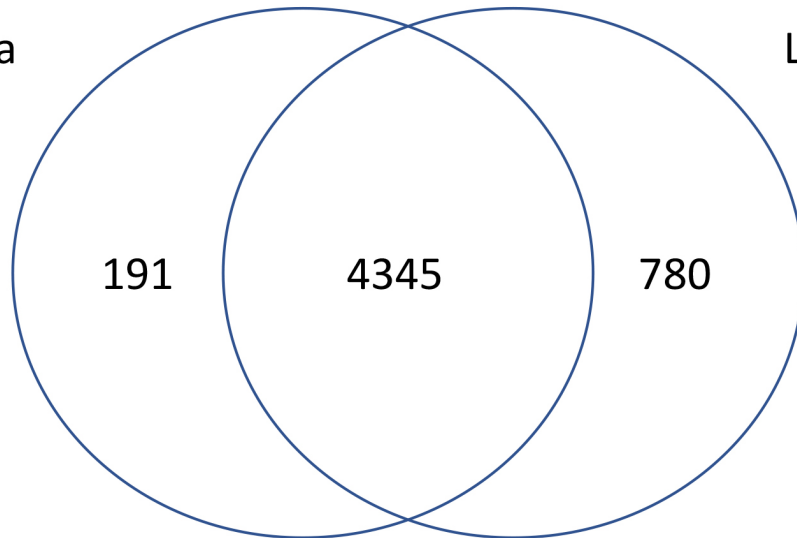

Supplement: FIG S3 [file mSystems.00216-19-sf003.pdf]

**A**

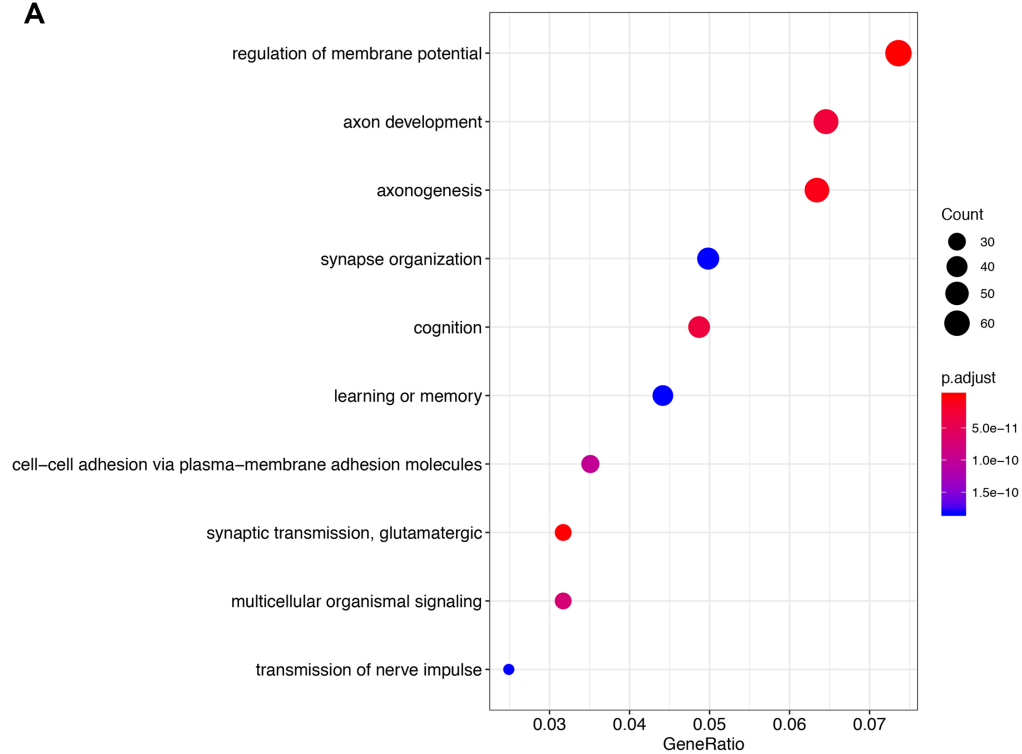

**B**

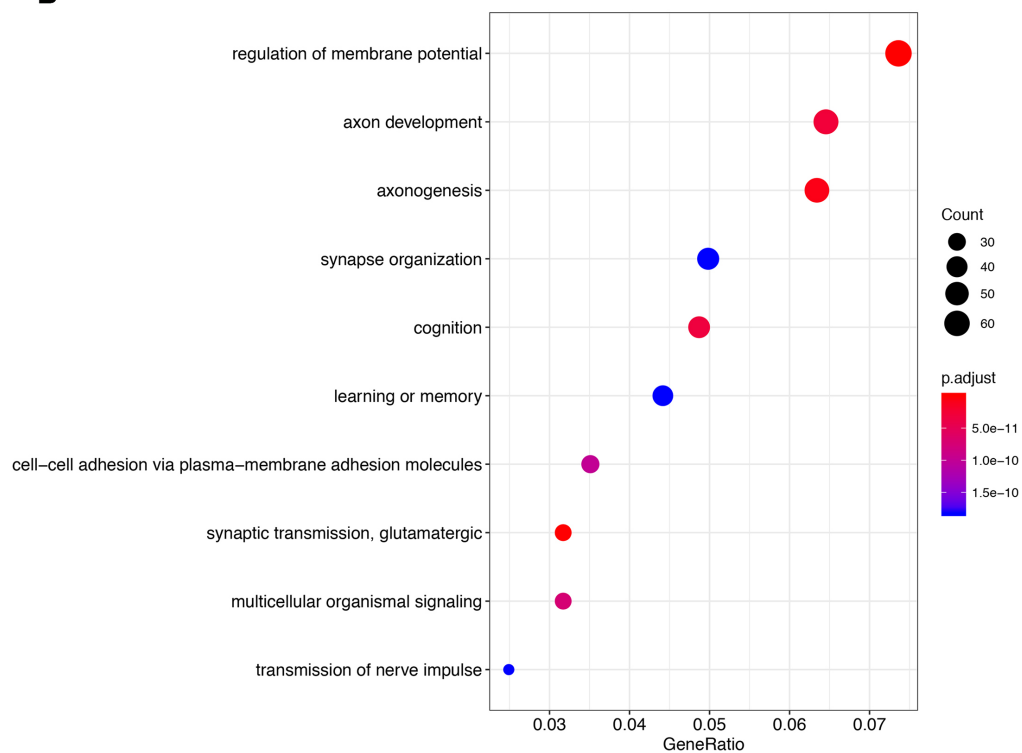

Supplement: FIG S4 [file mSystems.00216-19-sf004.pdf]

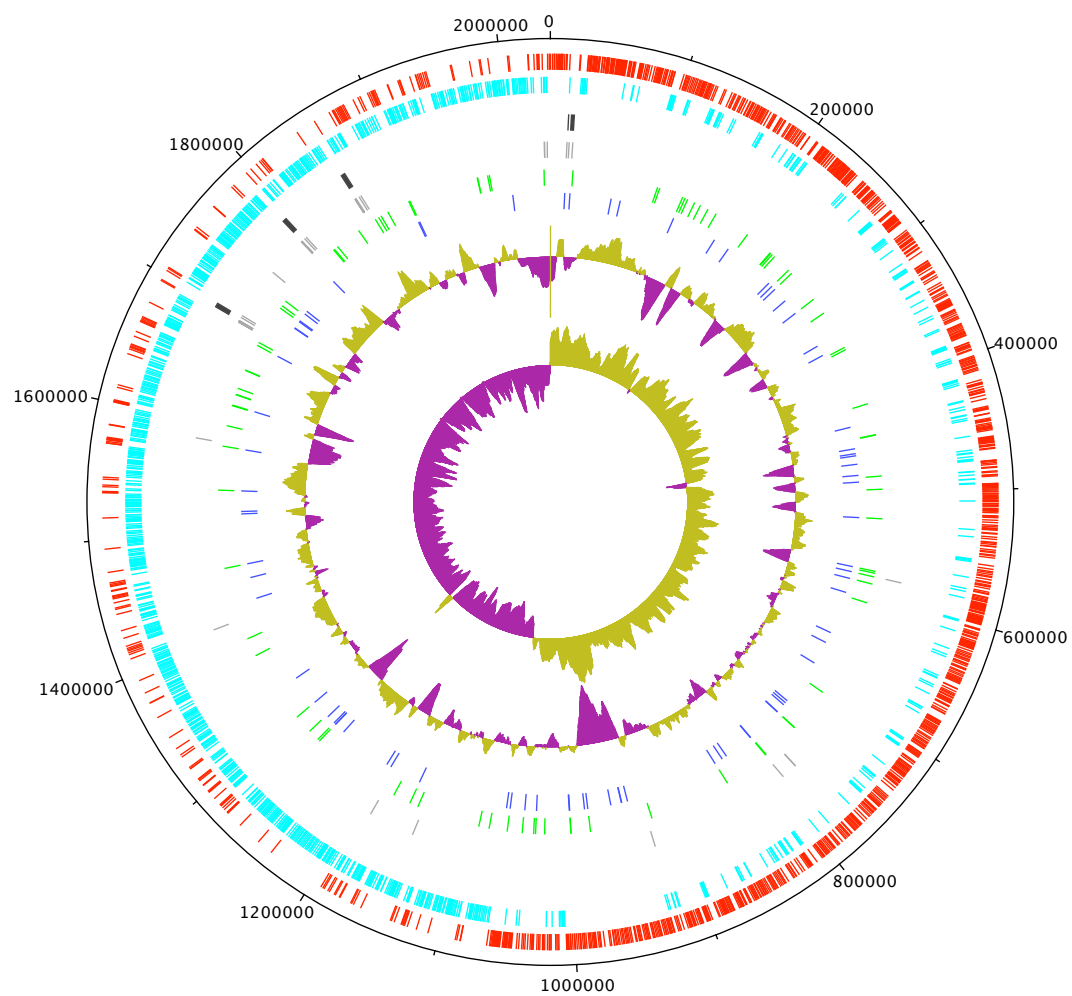

Supplement: FIG S5 [file mSystems.00216-19-sf005.pdf]

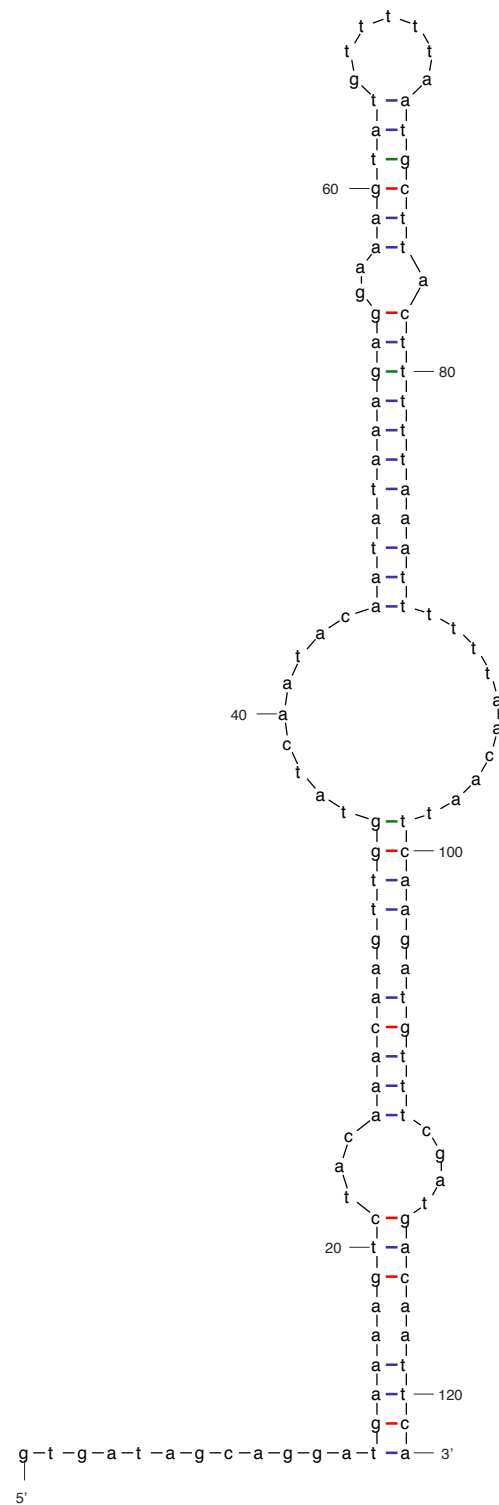

$dG = -17.9$  S 47

Supplement: FIG S6 [file mSystems.00216-19-sf006.pdf]
